# A Novel Approach to Monitor the Concentration of Phosphate Buffers in the Range of 1 M to 0.1 M Using a Silicon-Based Impedance Sensor

**DOI:** 10.3390/bios13090841

**Published:** 2023-08-24

**Authors:** Vinayak J. Bhat, Daniel Blaschke, Elke Müller, Ralf Ehricht, Heidemarie Schmidt

**Affiliations:** 1 Leibniz Institute of Photonic Technology, Albert-Einstein-Str. 9, 07745 Jena, Germany; vinayak-jayram.bhat@leibniz-ipht.de (V.J.B.); daniel.blaschke@leibniz-ipht.de (D.B.); ralf.ehricht@leibniz-ipht.de (R.E.); 2 Institute of Solid State Physics, Friedrich Schiller University Jena, Helmholtzweg 3, 07743 Jena, Germany; 3 Institute of Physical Chemistry, Friedrich Schiller University Jena, Helmholtzweg 4, 07743 Jena, Germany; mueller.elke@uni-jena.de; 4 InfectoGnostics Research Campus, 07743 Jena, Germany

**Keywords:** PolCarr^®^ impedance biochip, concentration, equivalent circuit modeling, impedance spectroscopy, phosphate buffer

## Abstract

We present a novel and easy approach using a silicon-based impedance chip to determine the concentration of the given aqueous buffer solution. An accurate determination of the post-dilution concentration of the buffers is necessary for ensuring optimal buffer capacity, pH stability, and to assess solution reproducibility. In this study, we focused on phosphate buffer as the test liquid to achieve precise post-dilution concentration determinations. The impedance chip consisting of a top gold ring electrode, where a test volume of 20 μL to 30 μL of phosphate buffer was introduced for impedance measurements within the frequency range of 40 Hz to 1 MHz. For impedance investigation, we used phosphate buffers with three different pH values, and the impedance was measured after diluting the phosphate buffers to a concentration of 1.00 M, 0.75 M, 0.50 M, 0.25 M, 0.10 M, 0.05 M, and 0.01 M. In order to analyze the distinctive changes in the measured impedance, an equivalent circuit was proposed and modeled. From the impedance modeling, we report that the circuit parameter R_Au/Si_ showed exponential dependence on the concentration of phosphate buffer and no dependence on the pH values of the phosphate buffer and on the added volume inside the ring electrode. The proposed silicon-based impedance chip is quick and uses reduced liquid volume for post-dilution concentration measurements of buffers and has perspective applications in the pharmaceutical and biological domains for regulating, monitoring, and quality control of the buffers.

## 1. Introduction

The knowledge of the exact concentration of the buffer solution is essential for precise pH regulation, accurate analytical methods, reliable biological and biochemical experiments [1,2], and pharmaceutical industrial applications [3]. It ensures reproducibility, reliable results, and desired optimal conditions in various fields of study. Additionally, precise post-dilution concentration assessment is crucial in toxicology [4,5,6] and clinical pharmacology [7,8] in ensuring safety and mitigating the risk associated with the substance being administered. Various methodologies have been employed to determine the concentration of diluted liquids, including concentration measurements [9], volume-based measurements [10], and dilution series measurements. Concentration-based measurements often rely on spectrophotometry, an analytical technique that quantitatively determines the concentration of a substance in a test solution by measuring its absorption or transmission of light using a transparent cuvette [11]. Spectrophotometry offers distinct advantages as a non-invasive and non-destructive technique, minimizing sample preparation requirements while enabling extensive large-scale quantitative analysis for concentration determination [12]. However, as pointed out by A. G. Reule [13], spectrophotometry can be prone to errors due to various spectral characteristics of the instrument. Additionally, this technique has limitations in terms of its sensitivity to low concentrations. On the other hand, volume-based dilution measurements, such as titrimetric analysis, involve the reaction between a known titrant and an unknown titrand solution. Although titrimetric analysis is widely used because of its simple, cost-effective, and straightforward approach, it also exhibits drawbacks related to the considerable time needed to prepare standards and titrants and has limitations in the resulting precision and liquid volume prerequisites for the measurement. Limitations of the existing techniques to determine the concentration of diluted liquids pose challenges in achieving real-time, accurate, and continuous monitoring and control of the post-dilution concentration of liquids in dynamic environments.

In this study, we introduce a pn-junction-based boron-implanted silicon impedance chip featuring a ring top electrode. The impedance chip operates on the principle of measuring the electrical impedance changes as a means to determine the concentration of the buffer solution. This approach offers an easy, rapid, and non-invasive means to determine the concentration after dilution, i.e., the post-dilution concentration. The presented impedance chip has several advantages compared to traditional concentration measurement techniques. Notably, the proposed method requires a significantly reduced volume of liquid, typically ranging from 20 μL to 30 μL, as opposed to substantial volumes of 0.4 mL to 3.5 mL for spectrophotometry [14] and 10 mL to 20 mL for titrimetric analysis techniques [15]. Furthermore, the impedance chip eliminates the need for reagents and offers relatively short measurement times, typically around 1 min. Additionally, there exists the potential for the integration of impedance chips within the inline buffer dilution configuration. The impedance chip can serve as a concentration measurement sensor integrated into the control monitoring system part of the inline dilution along with the existing pH and conductivity sensors [16] to comprehensively monitor the characteristics of the diluted solution.

To assess the efficacy of the impedance chip for dilution measurement, we utilized a phosphate buffer as the test liquid and determined the dilution concentration through impedance measurements with our impedance chip. The impedance measurements were conducted by introducing a small volume of the test liquid into the defined ring electrode on the impedance chip, followed by impedance measurement. For characterization purposes, we employed phosphate buffer solutions of different concentrations with three different pH values and measured the impedance for each pH value by gradually increasing the volume of phosphate buffer from 20 μL to 30 μL inside the ring electrode. This approach allowed us to evaluate the impedance chip’s performance across different pH conditions as well as with different volumes. The pn junction silicon impedance chip has previously been applied in bacterial cell counting applications [17,18]. Furthermore, Poltorak et al. [19] have utilized the doped silicon chips in investigating the lipid vesicles and polyelectrolyte interactions after impedance measurements in the frequency range from 100 kHz down to 100 mHz. This technique of electrical analysis using a silicon-based impedance sensor is used in the detection of real-time viability of a single cell after assessing the impedance change of a single chip in an integrated electrode array [20] and Chen Y. et al. [21] similarly applied an impedance sensor within the microelectrode array for counting and analysis of breast tumor cells within the frequency range of 100 Hz to 1 MHz. The current study provides a potential extended application of the silicon impedance sensor in the continuous determination and monitoring of buffer concentration.

## 2. Materials and Methods

### 2.1. Sodium Phosphate Buffer Preparation and Characterization

In this work, an aqueous sodium phosphate buffer is used as the test liquid for post-dilution concentration measurements. The phosphate buffer is prepared using Sorenson’s buffer method with varying ratios of 1 M sodium dihydrogen phosphate (NaH_2_PO_4_) and 1 M disodium hydrogen phosphate (Na_2_HPO_4_). Table 1 presents the ratios of NaH_2_PO_4_ and Na_2_HPO_4_ stock solutions used to obtain 100 mL of phosphate buffer with desired pH values. The corresponding experimental pH values, determined using a Schott CG 842 pH tester at 22 °C with probe InLab Micro from Mettler Toledo, are also provided in Table 1. For dilution measurements, three undiluted aliquots of phosphate buffer with pH values of 5.50 (Aliquot 1), 6.38 (Aliquot 2), and 7.12 (Aliquot 3) were selected. Deionized water (DI water) with a pH of 7.45 was added in corresponding amounts to each phosphate buffer aliquot, resulting in concentrations of 0.75 M, 0.50 M, 0.25 M, 0.10 M, 0.05 M, and 0.01 M for the respective aliquots. In Table 2, the dilution concentration and the corresponding experimentally determined pH values after dilution, using Schott CG 842 pH tester at 22 °C, are presented.

In order to confirm the increasing pH tendency with dilution, a secondary experiment was carried out by diluting the selected phosphate buffer aliquots with DI water of pH 5.56, sourced from the Milli-Q EQ 7000 Water Purification System. The obtained pH values are also tabulated in Table 2.

**Figure 1:**
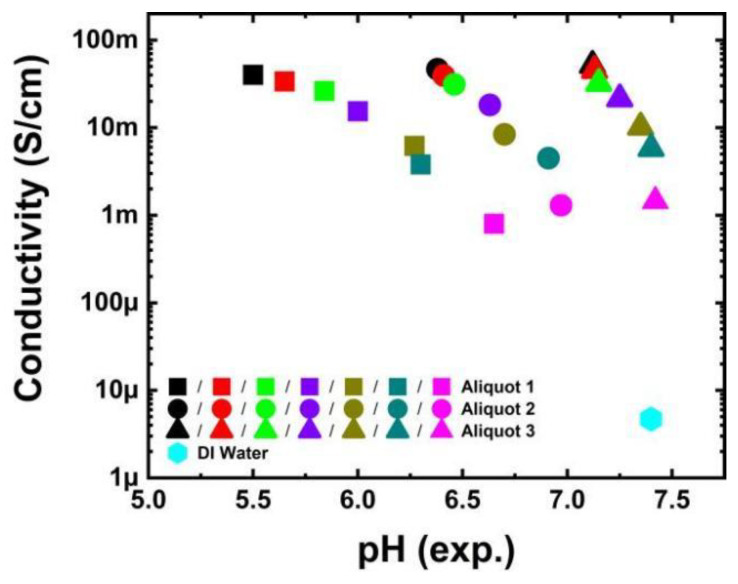
Conductivity vs. experimental pH values of the undiluted phosphate buffer (black dots), and diluted phosphate buffer with concentrations of 0.75 M (red), 0.50 M (green), 0.25 M (violet), 0.10 M (dark yellow), 0.05 M (dark cyan), and 0.01 M (magenta) are shown for the liquids in Aliquot 1 (square dots), Aliquot 2 (circle dots), and Aliquot 3 (triangle dots). The conductivity of the water is 3.4 μS/cm and is represented in a diamond dot.

The electrical conductivity of the phosphate buffer was measured using the Schott Lab 970 conductivity measurement instrument at a temperature of 21 °C. The obtained conductivity values for undiluted phosphate buffer, diluted phosphate buffer, and DI water are presented in Figure 1. The 1 M phosphate buffer exhibited a conductivity range of 40 mS/cm to 52 mS/cm for all three aliquots, which gradually decreased with dilution to a range of 0.8 mS/cm to 1.5 mS/cm for a phosphate buffer concentration of 0.01 M, consistent with theoretical expectations [22]. The conductivity of the DI water was determined to be 3.4 µS/cm.

### 2.2. Silicon-Based Impedance Sensor

In the investigation of dilution phosphate buffer solution, a boron-implanted silicon-based impedance chip (BG) was prepared. During the fabrication process of BG impedance chips, boron ions (B+) were implanted into 4-inch silicon-doped phosphorus (Si:P) wafers. Subsequently, a 150 nm thick gold (Au) ring top electrode with an inner diameter of 5.7 mm and an outer diameter of 7.8 mm, as well as an unstructured Au bottom electrode were deposited using dc-magnetron sputtering. The 4-inch wafers were then cut into 1 × 1 cm^2^ pieces. The implantation parameters and doping density are provided in our previous work [17]. Later, these impedance chips were treated with HF-dip to maintain the same water contact angle at the surface of the impedance chips. The ring electrodes of impedance chips prepared in this way have the capability to hold a liquid volume of up to 30 µL without any overflow.

### 2.3. Impedance Spectroscopy

**Figure 2:**
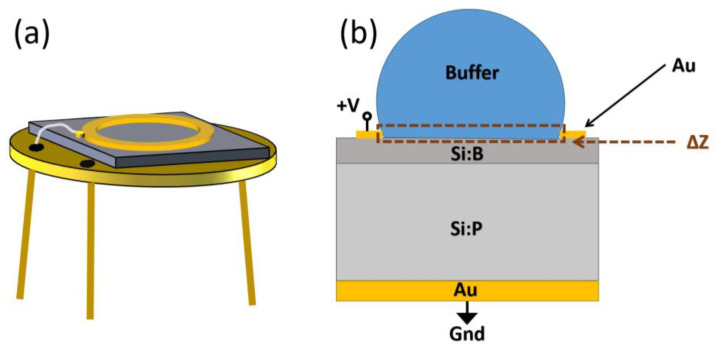
(**a**) Schematic representation of the boron-implanted pn-junction-based impedance chip, which includes a top gold (Au) ring electrode and a bottom gold electrode wire bonded to a TO-5 package. (**b**) Cross-sectional view of the impedance chip after filling the ring electrode with the phosphate buffer. Filling-dependent impedance change is depicted in the figure and represented as ΔZ.

The frequency-dependent impedance measurements were conducted using an Agilent 4294 impedance analyzer, in the frequency range of 40 Hz to 1 MHz, for both the empty impedance chip and the impedance chip filled with phosphate buffer inside the ring electrode. The temperature was maintained at 21 °C during the measurements, and each impedance measurement took approximately 1 min to record 100 data points within the frequency range. The impedance measurements were performed in the absence of light (dark condition). A schematic diagram of the impedance chip is presented in Figure 2, which includes the top view of the chip consisting of a top gold ring electrode, Si pn junction, and bottom gold electrode wire bonded to a TO-5 socket, shown in Figure 2a.

The cross-sectional schematic of the impedance chip with phosphate buffer liquid inside the ring electrode is shown in Figure 2b, where the height of the liquid depicted is much greater than the ring electrode height. The absolute value of the measured complex impedance of the impedance chip is plotted (Im (Z) vs. Re (Z)) and analyzed. The measured absolute impedance values for the three aliquots of the dilution are shown in the Supplementary Information. The boron-implanted impedance chip has an impedance range from 35 kΩ at 40 Hz to 10 Ω at 1 MHz for both empty and filled cases. Using the equivalent circuit model, we analyzed the changes in the complex impedance measured for empty, and after filling phosphate buffer inside the ring electrode. The filling-dependent complex impedance analyzed is denoted as ΔZ in Figure 2b.

### 2.4. Impedance Modeling

**Figure 3:**
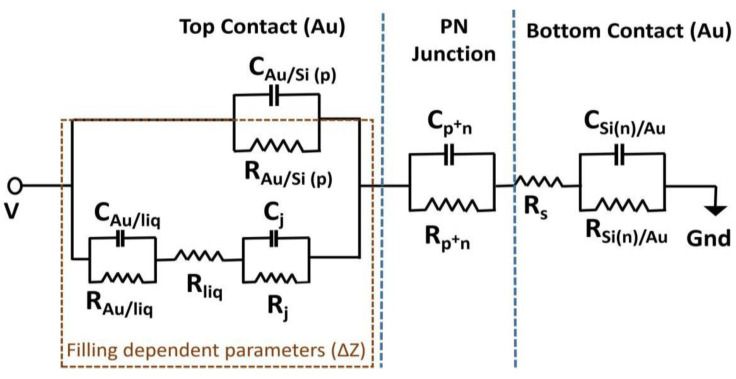
The equivalent circuit model of a boron-doped silicon impedance chip used to model measured impedance of empty and filled boron-doped silicon impedance chips. All the parameters for empty and for 20 μL phosphate-buffer-filled silicon impedance chips are modeled. Parameters within the highlighted box (R_Au/Si_, R_liq_, C_Au/liq_, R_Au/liq_, C_j_, and R_j_) are modeled for silicon impedance chips filled with more volume of phosphate buffer (22 μL to 30 μL) and other parameters are fixed to the modeled value with a filling of 20 μL. The three regions of the impedance chip are separated by the blue dotted lines. The left region represents the top contact gold and Si(p+) interface, the center represents the pn junction region, and the right region represents bottom contact between Si(n) and the gold electrode.

To determine the relationship between the measured changes in impedance due to the dilution of the added phosphate buffer, an equivalent circuit is considered based on the geometry of the sample and modeled. Figure 3 shows the schematic of the equivalent circuit which is divided into three regions. The rightmost region consists of the capacitance and resistance pairs C_Si(n)/Au_ and R_Si(n)/Au_, respectively, which represent the interface between the n-Si and the bottom gold contact. The center region contains the capacitance and resistance pairs C_p+n_ and R_p+n_, respectively, which describe the p+n junction of the sample. The leftmost region consists of the capacitance and resistance parameter pairs C_Au/Si(p+)_ and R_Au/Si(p+)_, respectively, representing the top contact gold ring electrode and the p-Si interface. The region also includes the capacitance and resistance parameter pairs C_j_ and R_j_, respectively, representing the junction of top gold contact, p-Si, and the phosphate buffer liquid once the phosphate buffer is filled inside the ring electrode. The capacitance and resistance parameter pairs C_Au/liq_ and R_Au/liq_, respectively, describe the capacitance and resistance values between the top gold electrode and the liquid, while R_liq_ describes the resistance between the phosphate buffer and the p-Si.

To establish the initial modeling parameter values, the capacitance values of the top contact and p-Si interface (C_Au/Si_), the p+n junction capacitance (C_p+n_), and the n-Si and bottom contact interface capacitance (C_Si(n)/Au_) are calculated. These calculated capacitance values are used as start values for determining the remaining parameters for the empty impedance chip. The equations used to calculate the capacitance are as follows:

(1) $$C_{p+n}=\frac{\varepsilon \varepsilon_{0}A}{x_{d}}$$

(2) $$C_{\mathrm{Au}/\mathrm{Si}(p)}=A\cdot \sqrt{(q\varepsilon \varepsilon_{0}N_{A})/2\Phi_{bi,p+}}$$

(3) $$C_{\mathrm{Si}(n)/\mathrm{Au}\;}=\;A\cdot \sqrt{(q\varepsilon \varepsilon_{0}N_{D})/2\Phi_{bi,n}}$$

The relative permittivity ε of silicon is 11.7, the permittivity of free space ε_0_ is 8.854 × 10^−12^ F/m, the depletion width is calculated to be 628 nm, and area *A* of the top electrode is 22.3 mm^2^. The built-in potential value of the top gold ring electrode and Si(p+) interface Φ_bi,p+_ is 0.19 eV, and of Si(n) and gold bottom contact is Φ_bi,n_ is 1.05 eV. The carrier concentration values of N_A_ and N_D_ are 400 × 10^15^ cm^−3^ and 2.5 × 10^15^ cm^−3^, respectively. The carrier concentration and built-in potential are referred from our previous work [18]. After filling 20 μL of phosphate buffer with a pipette inside the ring electrode, the values are varied in the similar range to model the total impedance of the system once the sample reaches equilibrium with the phosphate buffer liquid. We can represent the system impedance after filling 20 μL of phosphate buffer as Z_system_ equals to Z_20 μL_. In the subsequent steps, when +2 μL is added with a pipette, the change in system impedance can be described as Z_system_ = Z_20 μL_ + ΔZ, where ΔZ includes the changes in the filling-dependent impedance parameters, namely C_Au/liq_, R_Au/liq_, C_j_, R_j_, R_liq_, and R_Au/Si_ from the equivalent circuit model. These filling parameters are adjusted upon the successful addition of +2 μL of phosphate buffer to the initial 20 μL volume inside the ring electrode.

## 3. Results and Discussion

**Figure A1:**
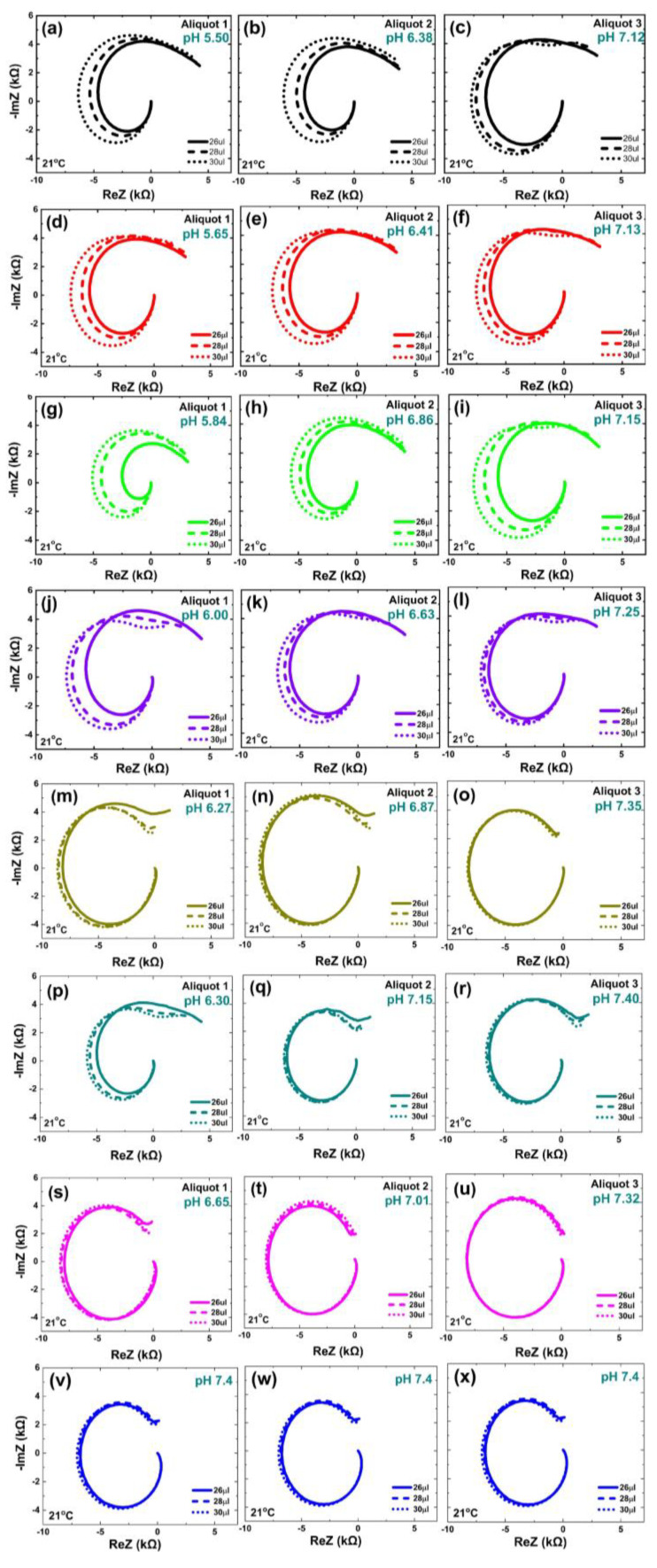
Nyquist plot of the extracted impedance measurements of the undiluted phosphate buffer (**a**–**c**), with dilution concentrations of 0.75 M (**d**–**f**), 0.50 M (**g**–**i**), 0.25 M (**j**–**l**), 0.10 M (**m**–**o**), 0.05 M (**p**–**r**), and 0.01 M (**s**–**u**) for Aliquot 1, 2, and 3 is shown. The extracted impedance for the DI water is shown in (**v–x**) for comparison. The convention of the color taken for the undiluted and all the dilutions is kept the same in the figure. The added liquid amount 26 μL inside the ring electrode is represented in a solid line, 28 μL is represented in a dashed line, and 30 μL is represented in a dotted line. All the impedance measurements are performed at 21 °C.

For impedance measurements using the boron-doped Si impedance chip, separate impedance chips were used for each dilution of phosphate buffer samples in Aliquot 1, Aliquot 2, and Aliquot 3 measurements. Initially, an empty chip, without phosphate buffer inside the ring electrode, was measured within the specified frequency range. In the next step, 20 µL of phosphate buffer from the respective test aliquot was pipetted out and filled inside the top ring electrode of the impedance chip. The impedance measurement was then performed within the frequency range after the phosphate buffer reached equilibrium with the impedance chip, typically taking approximately ~5–6 min. Following that, +2 μL of the same test aliquot was added inside the ring electrode in five consecutive steps, and the impedance data were recorded after each addition. To ensure the validation of the comparison between the measured impedance of the three test aliquots on different impedance chips, we have extracted the impedance of the filled measurements by subtracting the real and imaginary values of the empty measurements from the corresponding real and imaginary part of the measurement after adding +2 µL, +2 μL, +2 μL, +2 μL, and +2 μL to the 20 µL phosphate buffer inside ring electrode in the subsequent steps. The Nyquist plot of extracted impedance data for undiluted and diluted phosphate buffer for total volumes of 26 μL, 28 μL, and 30 μL inside the ring electrode is shown in Figure A1.

The Impedance chip is sensitive to the change in dilution, pH, and volume. The extracted impedance values depicted in Figure A1 illustrate the combined changes observed in the real and imaginary components of the Nyquist plot. Notably, there is an increase in the extracted impedance values for undiluted phosphate buffer and diluted phosphate buffer samples with concentrations of 0.75 M, 0.50 M, and 0.25 M as the volume inside the ring electrode increases. Conversely, dilutions with concentrations of 0.10 M, 0.05 M, and 0.01 M exhibit minimal changes and are comparable to the impedance changes observed when adding DI water (blue) inside the ring electrode. These changes are effectively captured and modeled by changing the filling parameters ΔZ of the equivalent circuit model.

**Figure 4:**
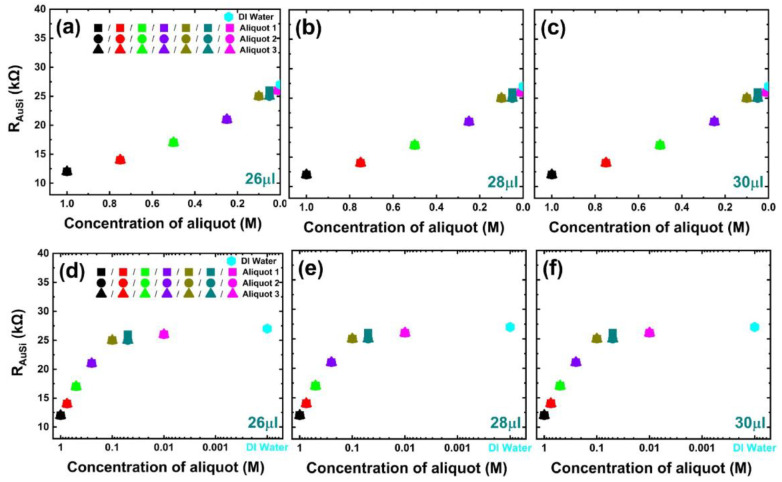
Modeled equivalent circuit parameter R_Au/Si_ versus molar concentration of Aliquot 1 (pH 5.50), Aliquot 2 (pH 6.38), and Aliquot 3 (pH 7.12) plotted (**a**–**c**) on a linear scale and (**d**–**f**) log scale for a liquid volume of (**a**,**d**) 26 μL, (**b**,**e**) 28 μL, and (**c**,**f**) 30 μL. Modeled R_Au/Si_ does not depend on the liquid volume and also does not depend on the pH value, the corresponding symbols overlap. Modeled R_Au/Si_ for pure DI water is represented with a diamond dot and has a value of 27 kΩ.

The obtained modeled circuit parameter values for the empty impedance chip and the phosphate-buffer-filled impedance chip with a volume of 26 μL are presented in Appendix B Table A1, Table A2 and Table A3. Among the filling-dependent parameters, the resistance R_Au/Si_, which represents the Schottky contact between the top contact and silicon interface, exhibits a systematic dependency on the concentration of the phosphate buffer liquid. Figure 4a–c illustrates the linear-scale plot of R_Au/Si_ against the concentration of the phosphate buffer solution, while Figure 4d–f shows the logarithmic-scale plot.

The overlapping of the three dots (in Figure 4) representing Aliquot 1, Aliquot 2, and Aliquot 3 indicates that the circuit parameter R_Au/Si_ is independent of the pH value of the phosphate buffer and remains consistent for added volumes of 26 μL, 28 μL, and 30 μL inside the top ring electrode, suggesting its independence from the volume as well. We observe two regions with a different slope (region I: 1 M < concentration < 0.1 M, region II: 0.1 M < concentration < 0). In region I, R_Au/Si_ shows a linear behavior on a semi-logarithmic plot and in region II, R_Au/Si_ is nearly constant. Therefore, R_Au/Si_ has an exponential dependence on the phosphate buffer concentration in region I. To quantify the correlation R_Au/Si_ vs. concentration, we used an exponential function and related the concentration C of the diluted buffer to the modeled R_Au/Si_ with b representing rate, A as initial value, and R_0_ as offset.

$$C=\frac{1}{b}\;\ln\;\frac{R_{Au/Si}-R_{0}}{A}$$

**Figure 5:**
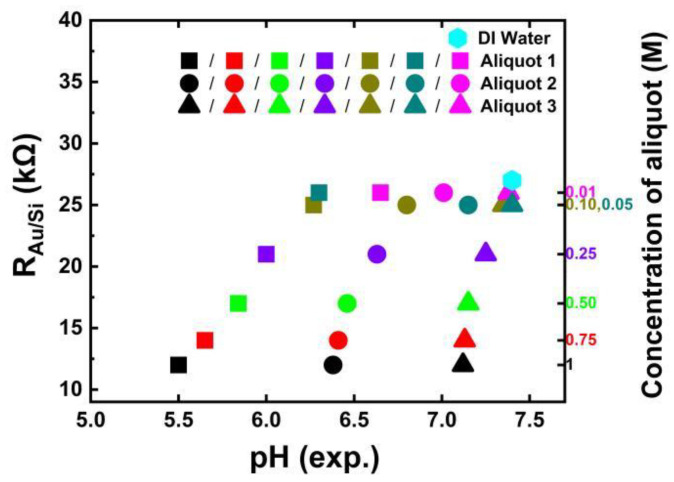
Modeled equivalent circuit parameter R_Au/Si_ versus experimentally determined pH values of Aliquot 1, Aliquot 2, and Aliquot 3 plotted on a linear scale for the liquid volume of 26 μL. Modeled R_Au/Si_ for pure DI water is represented with a diamond dot and has a value of 27 kΩ.

**Figure A2:**
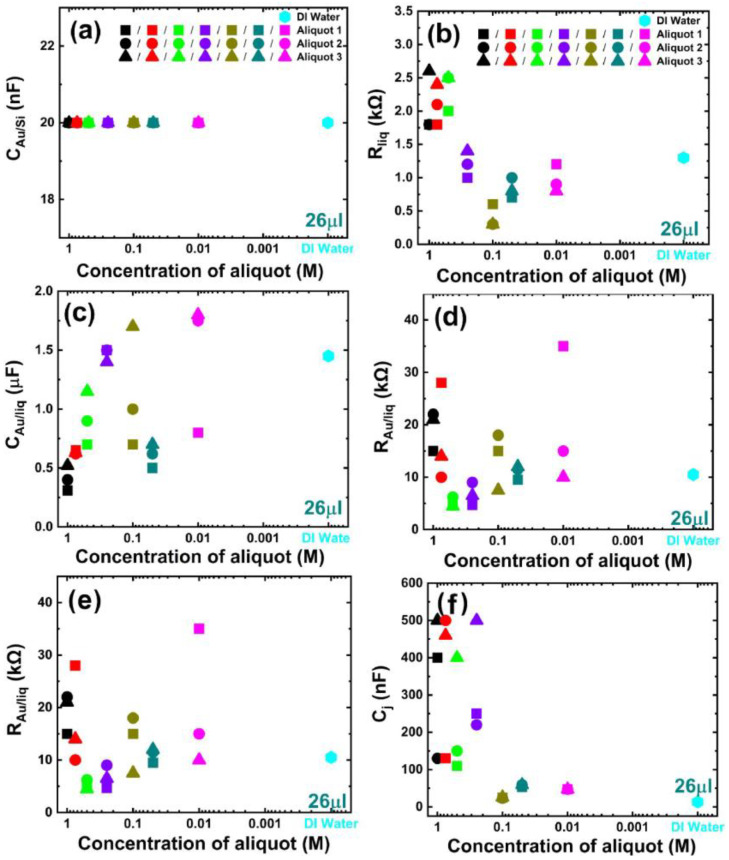
Modeled equivalent circuit parameters (**a**) C_Au/Si_, (**b**) R_liq_, (**c**) C_Au/liq_, (**d**) R_Au/liq_, (**e**) C_j_, and (**f**) R_j_ against the dilution concentrations of phosphate buffer test liquid for the added volume of 26 μL inside the top ring electrode. Parameter C_Au/Si_ shows no dependency on dilution, volume, and pH, whereas other filling parameters are varied with dilution, pH, and added volume. The phosphate buffer Aliquot 1 is represented in a square dot, Aliquot 2 in a circle dot, and Aliquot 3 in a triangle dot, respectively.

The R_Au/Si_ vs. concentration fitting is shown in Figure S2 of the Supplementary Information. The remaining filling parameters, namely C_j_, R_j_, C_Au/liq_, R_Au/liq_, and R_liq_, exhibit dependencies on both pH and volume. Appendix C Figure A2 presents the modeling results for these filling-dependent parameters. Figure 5 shows the R_Au/Si_ change with the experimentally determined pH values of the different concentrated phosphate buffers in Aliquot 1, Aliquot 2, and Aliquot 3. The value of the R_Au/Si_ parameter varies from 12 kΩ for undiluted phosphate buffer to 26 kΩ for a dilution of 0.01 M of phosphate buffer, while the R_Au/Si_ value for DI water is 27 kΩ.

In summary, the impedance chip offers improved precision and real-time monitoring the concentration of diluted liquids, and the results of this study are helpful, particularly in the inline buffer dilution configuration, where the impedance chip can be integrated into the second stage of the inline dilution, the control monitoring unit, or inline feedback control. After combining the desired concentrated buffer solution and the water from the two inlet streams in a mixer module, the diluted solution flows through the impedance chip. The impedance change information can be relayed back to the computer for real-time monitoring of concentration in the diluted liquid by checking the modeled circuit parameter R_Au/Si_ to confirm the buffer dilution range.

## 4. Conclusions

In the present work, we propose a novel approach to determine the concentration of the phosphate buffer using boron-doped silicon impedance chips with a ring top electrode. The impedance chip demonstrates sensitivity to liquid concentration change and offers significant advantages over other standard concentration measurement techniques. We have measured and analyzed the impedance response of the phosphate buffer of three different pH values with concentrations of 1.00 M, 0.75 M, 0.50 M, 0.25 M, 0.10 M, 0.05 M, and 0.01 M. The pH value of the undiluted (1.00 M) phosphate buffer was determined to be 5.50, 6.38, and 7.12, and showed a slight increase with dilution. The obtained impedance results were then subsequently analyzed with the developed equivalent circuit model which related to the observed changes in the impedance due to dilution, pH, and volume. From the modeling, the circuit parameter R_Au/Si_ showed dependence on diluted concentration change while being independent of the change in volume and pH of the phosphate buffer test liquid inside the ring electrode. The impedance chip offered improved sensitivity, reduced liquid volume requirements, and faster measurement times (approximately 1 min) compared to traditional techniques. The presented work is shown for the aqueous phosphate buffer; in the future, we intend to investigate a broader range of buffers, thereby enhancing the resolution of equivalent circuit parameters and exploring the application of impedance chips in microbiology and the food industry.

## Figures and Tables

**Table 1:** Phosphate buffer preparation table to obtain different pH values from combining di-sodium phosphate and sodium dihydrogen phosphate solutions using Sorenson’s buffer method to obtain a 100 mL solution.

1 M NaH_2_PO_4_ (mL)	1 M Na_2_HPO_4_ (mL)	pH (Experiment)
80	20	5.50 (Aliquot 1)
50	50	6.38 (Aliquot 2)
20	80	7.12 (Aliquot 3)

**Table 2:** Experimental pH values of phosphate buffer test liquid with concentrations of 1.00 M, 0.75 M, 0.50 M, 0.25 M, 0.10 M, 0.05 M, and 0.01 M phosphate buffer in Aliquot 1 (initial pH = 5.50), Aliquot 2 (initial pH = 6.38), and Aliquot 3 (initial pH = 7.12). For comparison, the phosphate buffer was diluted with DI water of pH 7.45 and DI water of pH 5.56.

Aliquot	Concentration of Aliquot (M)	pH (Experiment) DI Water pH 7.45	pH (Experiment) DI Water pH 5.56
Aliquot 1	1.00	5.50	5.67
	0.75	5.65	5.76
	0.50	5.84	5.91
	0.25	6.00	6.10
	0.10	6.27	6.30
	0.05	6.30	6.40
	0.01	6.65	6.55
Aliquot 2	1.00	6.38	6.42
	0.75	6.41	6.53
	0.50	6.46	6.61
	0.25	6.63	6.71
	0.10	6.70	6.89
	0.05	6.93	6.96
	0.01	6.97	7.15
Aliquot 3	1.00	7.12	7.18
	0.75	7.13	7.31
	0.50	7.15	7.32
	0.25	7.25	7.40
	0.10	7.35	7.52
	0.05	7.40	7.59
	0.01	7.38	7.75

## Data Availability

The data that support the findings of this study are available from the corresponding author, H.S., upon reasonable request.

## Supplementary Materials

The following supporting information can be downloaded at: https://www.mdpi.com/article/10.3390/bios13090841/s1, Figure S1: Nyquist plots of measured and modelled boron-doped Si impedance chip for empty and after filling 26 μL of phosphate buffer with various concentration (1.00 M, 0.75 M, 0.50 M, 0.25 M, 0.10 M, 0.05 M, and 0.01 M) inside the top ring electrode for (a) Aliquot 1, (b) Aliquot 2, and (c) Aliquot 3. Figure S2: Fitting of the circuit parameter R_Au/Si_ to the phosphate buffer concentration using an exponential growth fitting function. The function is applicable within the concentration range of 1.00 M to 0.10 M, while below 0.10 M concentration, the change in the value of R_Au/Si_ with the concentration becomes negligible.

## Appendix B

**Table A1:** Equivalent circuit parameter values for the empty impedance chip and corresponding values obtained for investigating phosphate buffer test liquid in Aliquot 1 with concentration of 1.00 M, 0.75 M, 0.50 M, 0.25 M, 0.10 M, 0.05 M, and 0.01 M. The obtained values are for the added liquid volume of 26 μL inside the top ring electrode of the impedance chip. During the equivalent circuit modeling of empty impedance, filling-dependent parameters are modeled with a cutoff frequency below 40 Hz to ensure no effect on the empty modeling.

26 μL	Top Contact							p+n Junction			Bottom Contact	
	C_Au/liq_ (μF)	R_Au/liq_ (kΩ)	Cj (nF)	Rj (kΩ)	R_liq_ (kΩ)	C_Au/Si(p+) _ (nF)	R_Au/Si(p+)_ (kΩ)	C_p+n_ (nF)	R_p+n_ (kΩ)	Rs (Ω)	C_Si(n)/Au_ (nF)	R_Si(n)/Au_ (Ω)
**Aliquot 1**												
Empty	20.00	0.30	160.00	2.80	2.25	16.00	11.00	5.90	25.70	7.00	17.00	14.00
1.00 M	0.31	15.00	400.00	2.20	1.80	20.00	12.00	8.00	27.30	8.00	17.00	26.00
Empty	20.00	0.30	160.00	2.80	2.25	16.00	11.00	5.90	25.70	7.00	17.00	14.00
0.75 M	0.65	28.00	130.00	10.50	1.80	20.00	14.00	9.00	24.80	8.00	15.00	29.00
Empty	20.00	0.30	160.00	2.80	2.25	16.00	11.00	5.90	28.00	7.00	17.00	14.00
0.50 M	0.70	5.20	110.00	7.50	2.00	20.00	17.00	6.70	27.80	8.00	16.00	29.00
Empty	20.00	0.30	160.00	2.80	2.25	16.00	8.00	5.80	23.40	6.00	16.00	15.00
0.25 M	1.50	4.70	250.00	6.00	1.00	20.00	21.00	8.70	25.40	8.00	10.00	53.00
Empty	20.00	0.30	160.00	2.80	2.25	16.00	11.00	5.90	25.70	7.00	17.00	14.00
0.10 M	0.70	15.00	25.00	2.80	0.60	20.00	25.00	12.00	25.80	9.00	8.00	55.00
Empty	20.00	0.30	160.00	2.80	2.25	16.00	11.00	5.90	15.55	7.00	17.00	14.00
0.05 M	0.50	9.50	53.00	8.80	0.70	20.00	26.00	10.20	16.90	7.00	11.00	80.00
Empty	20.00	0.30	160.00	2.80	2.25	16.00	11.00	5.90	18.00	7.00	17.00	14.00
0.01 M	0.80	35.0	47.00	11.80	1.20	20.00	26.00	12.00	19.00	7.00	11.00	80.00

**Table A2:** Equivalent circuit parameter values for the empty impedance chip and corresponding values obtained for investigating phosphate buffer test liquid in Aliquot 2 with concentration of 1.00 M, 0.75 M, 0.50 M, 0.25 M, 0.10 M, 0.05 M, and 0.01 M. The obtained values are for the added liquid volume of 26 μL inside the top ring electrode of the impedance chip. During the equivalent circuit modeling of empty impedance, filling-dependent parameters are modeled with a cutoff frequency below 40 Hz to ensure no effect on the empty modeling.

26 μL	Top Contact							p+n Junction			Bottom Contact	
	C_Au/liq _ (μF)	R_Au/liq _ (kΩ)	Cj (nF)	Rj (kΩ)	R_liq_ (kΩ)	C_Au/Si(p+) _ (nF)	R_Au/Si(p+) _ (kΩ)	C_p+n_ (nF)	R_p+n_ (kΩ)	Rs (Ω)	C_Si(n)/Au_ (nF)	R_Si(n)/Au_ (Ω)
**Aliquot 2**												
Empty	20.00	0.30	160.00	2.80	2.25	16.00	11.00	5.90	25.60	7.00	17.00	14.00
1.00 M	0.40	22.00	130.00	10.20	1.80	20.00	12.00	8.00	24.60	8.00	21.00	28.00
Empty	20.00	0.30	160.00	2.80	2.25	16.00	11.00	5.90	25.60	7.00	17.00	14.00
0.75 M	0.62	10.00	500.00	3.50	2.10	20.00	14.00	9.20	27.10	9.00	15.00	23.00
Empty	20.00	0.30	170.00	2.80	2.25	16.00	11.00	5.90	25.60	7.00	17.00	14.00
0.50 M	0.90	6.20	150.00	6.50	2.50	20.00	17.00	8.20	26.30	8.00	16.00	29.00
Empty	20.00	0.30	160.00	2.80	2.30	16.00	8.00	5.90	22.90	6.00	16.00	15.00
0.25 M	1.50	9.00	220.00	6.50	1.20	20.00	21.00	9.50	24.20	8.00	10.00	33.00
Empty	20.00	0.30	160.00	2.80	2.25	16.00	11.00	5.90	25.70	7.00	17.00	14.00
0.10 M	1.00	18.00	23.00	2.80	0.30	20.00	25.00	12.00	26.90	9.00	10.00	35.00
Empty	20.00	0.30	160.00	2.80	2.25	16.00	11.00	5.90	15.55	7.00	17.00	14.00
0.05 M	0.60	11.50	59.00	5.20	1.00	20.00	25.00	12.10	16.00	7.00	8.00	50.00
Empty	20.00	0.30	160.00	2.80	2.25	16.00	11.00	5.90	18.00	7.00	17.00	14.00
0.01 M	1.75	15.00	47.00	11.50	0.90	20.00	26.00	12.50	18.60	7.00	13.00	52.00

**Table A3:** Equivalent circuit parameter values for the empty impedance chip and corresponding values obtained for investigating phosphate buffer test liquid in Aliquot 3 with concentration of 1.00 M, 0.75 M, 0.50 M, 0.25 M, 0.10 M, 0.05 M, and 0.01 M. The obtained values are for the added liquid volume of 26 μL inside the top ring electrode of the impedance chip. During the equivalent circuit modeling of empty impedance, filling-dependent parameters are modeled with a cutoff frequency below 40 Hz to ensure no effect on the empty modeling.

26 μL	Top Contact							p+n Junction			Bottom Contact	
	C_Au/liq_ (μF)	R_Au/liq_ (kΩ)	Cj (nF)	Rj (kΩ)	R_liq_ (kΩ)	C_Au/Si(p+) _ (nF)	R_Au/Si(p+) _ (kΩ)	C_p+n_ (nF)	R_p+n_ (kΩ)	Rs (Ω)	C_Si(n)/Au_ (nF)	R_Si(n)/Au_ (Ω)
**Aliquot 3**												
Empty	20.00	0.30	160.00	2.80	2.25	16.00	11.00	5.90	24.40	7.00	17.00	14.00
1.00 M	0.52	21.00	500.00	6.50	2.60	20.00	12.00	10.60	24.80	9.00	17.00	18.00
Empty	20.00	0.30	160.00	2.80	2.25	16.00	11.00	5.90	24.40	7.00	17.00	14.00
0.75 M	0.63	14.00	460.00	3.90	2.40	20.00	14.00	10.30	25.20	8.00	15.00	33.00
Empty	20.00	0.30	170.00	2.80	2.25	16.00	11.00	5.90	25.40	7.00	17.00	14.00
0.50 M	1.15	4.50	400.00	4.80	2.50	20.00	17.00	9.60	26.30	8.00	16.00	48.00
Empty	20.00	0.30	160.00	2.80	2.30	16.00	8.00	5.90	22.70	6.00	16.00	15.00
0.25 M	1.40	6.50	500.00	5.00	1.40	20.00	21.00	10.50	23.60	8.00	12.00	34.00
Empty	20.00	0.30	160.00	2.80	2.25	16.00	11.00	5.90	25.70	7.00	17.00	14.00
0.10 M	1.70	7.50	26.00	2.80	0.30	20.00	25.00	12.30	26.60	9.00	10.00	50.00
Empty	20.00	0.30	160.00	2.80	2.25	16.00	11.00	5.90	15.55	7.00	17.00	14.00
0.05 M	0.70	12.00	59.00	5.20	0.80	20.00	25.00	11.80	17.00	7.00	8.00	80.00
Empty	20.00	0.30	160.00	2.80	2.25	16.00	11.00	5.90	18.00	7.00	17.00	14.00
0.01 M	1.80	10.00	47.00	11.80	0.80	20.00	26.00	12.00	20.10	7.00	12.00	40.00
