# Supplementary material for: A Novel Approach to Monitor the Concentration of Phosphate Buffers in the Range of 1 M to 0.1 M Using a Silicon-Based Impedance Sensor
Source: Biosensors (Basel). 2023 Aug 24;13(9):841. doi: 10.3390/bios13090841 (PMC10527345; doi:10.3390/bios13090841)
Supplement: Supplementary file 1 [file biosensors-13-00841-s001.zip › biosensors-2540951-supplementary.pdf]

biosensors

MDPI

Supplementary material

A Novel Approach to Monitor the Concentration of Phosphate Buffers in the Range of 1 M to 0.1 M Using a Silicon-Based Impedance Sensor

Vinayak J. Bhat [1,2], Daniel Blaschke [1], Elke Müller [3,4], Ralf Ehricht [1,3,4] and Heidemarie Schmidt [1,2,*]

[1] Leibniz Institute of Photonic Technology, Albert-Einstein-Str. 9, 07745 Jena, Germany; vinayak-jayram.bhat@leibniz-ipht.de (V.J.B.); daniel.blaschke@leibniz-ipht.de (D.B.); ralf.ehricht@leibniz-ipht.de (R.E.)
[2] Institute of Solid State Physics, Friedrich Schiller University Jena, Helmholtzweg 3, 07743 Jena, Germany
[3] Institute of Physical Chemistry, Friedrich Schiller University Jena, Helmholtzweg 4, 07743 Jena, Germany; mueller.elke@uni-jena.de
[4] InfectoGnostics Research Campus, 07743 Jena, Germany
* Correspondence: heidemarie.schmidt@leibniz-ipht.de

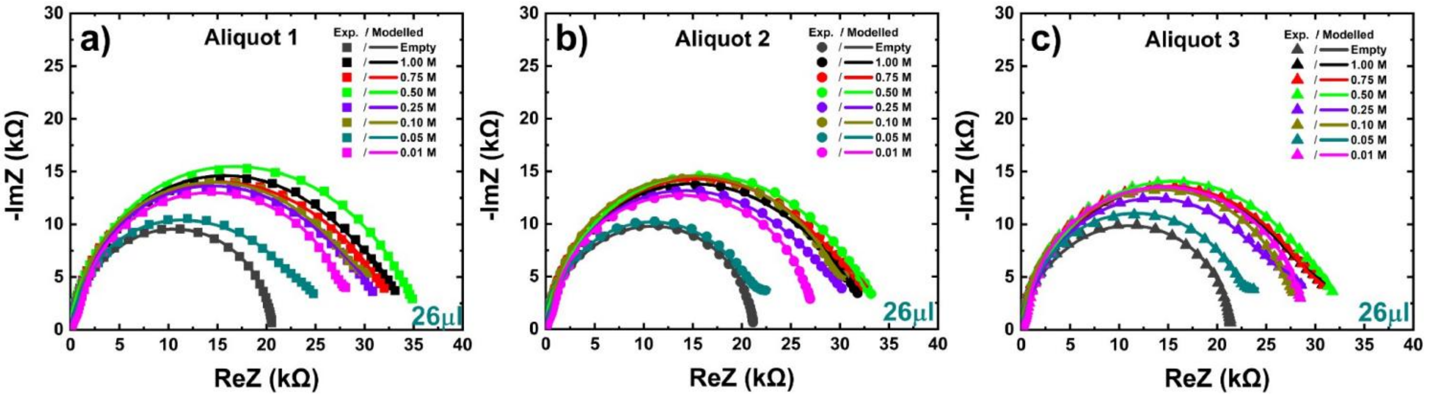

Figure S1. Nyquist plots of measured and modelled boron doped Si impedance chip for empty and after filling 26 µl of phosphate buffer with various concentration (1.00 M, 0.75 M, 0.50 M, 0.25 M, 0.10 M, 0.05 M, and 0.01 M) inside the top ring electrode for (a) Aliquot 1, (b) Aliquot 2, and (c) Aliquot 3. The measured data is denoted by dots, while the modelled results are represented by solid lines.

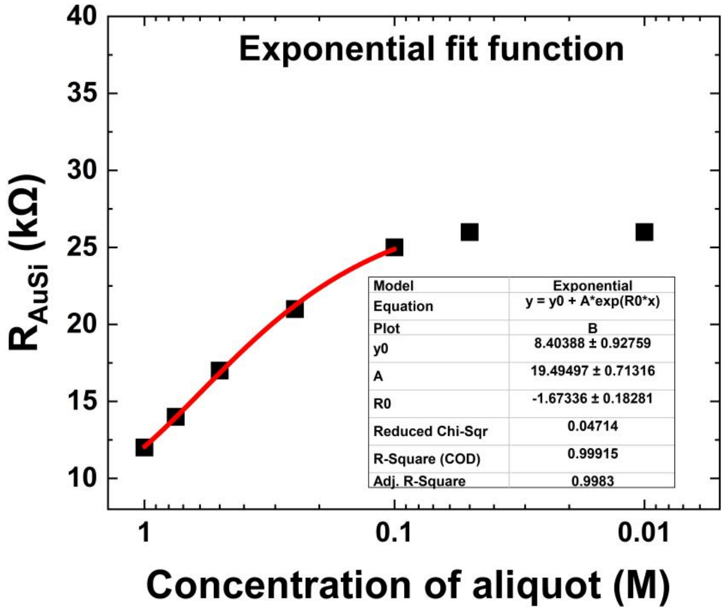

Figure S2. Fitting of the circuit parameter $R_{Au/Si}$ to the phosphate buffer concentration using an exponential growth fitting function. The function is applicable within the concentration range of 1.00 M to 0.10 M, while below 0.10 M concentration, the change in the value of $R_{Au/Si}$ with the concentration becomes negligible.
